# Consumption of vegetables and fruits and breast cancer survival: a systematic review and meta-analysis

**DOI:** 10.1038/s41598-017-00635-5

**Published:** 2017-04-04

**Authors:** Juanjuan He, Yuanting Gu, Shaojin Zhang

**Affiliations:** 1 Department of Breast Surgery, the First Affiliated Hospital, Zhengzhou University, Zhengzhou, Henan Province, China; 2 Department of Urology Surgery, the First Affiliated Hospital, Zhengzhou University, Zhengzhou, Henan Province, China

## Abstract

We aimed to conduct a meta-analysis investigating the association between consumption of vegetables and fruits and breast cancer survival. A comprehensive search of the PubMed and EMBASE was performed from the inception to September 30, 2016. The summary hazard ratios (HRs) and 95% confidence intervals (CIs) were estimated using a random effects model. Ten studies, with a total of 31,210 breast cancer cases, were included in the meta-analysis. The summary HRs (95% CIs) of overall survival (highest vs. lowest) were 1.08 (0.88–1.33; I^2^ = 41.1%) for pre-diagnostic intake of vegetables and fruits combined, 0.96 (0.71–1.30; I^2^ = 48.4%) for vegetables alone, and 0.83 (0.67–1.02; I^2^ = 0) for fruit alone. No significant risk associations of overall survival were found for post-diagnostic intake of vegetables and fruits. Line dose-response analyses indicated the likely results for both pre- and post- diagnostic dietary intake. No significant association was found between intake of vegetables and fruits and breast cancer-specific mortality. In addition, intake of cruciferous vegetables was not associated with death from breast cancer. Our findings indicated a borderline inverse association between pre-diagnostic intake of fruit and overall survival of breast cancer, whereas intake of vegetables was not associated with survival.

## Introduction

Breast cancer (BC) is the most commonly diagnosed cancer and the leading cause of cancer-related death in women worldwide. During the past 25 years, breast cancer death rates have been increasing in economically developing countries^1^: about 60% of the deaths are estimated to occur in those countries^1^. For a long time, researchers have focused on the beneficial effects of consumption of vegetables and fruits (VF) on the prevention of cancers, including breast cancer^2,3^. According to the 2007 WCRF/AICR Second Expert Report followed by the Updated Breast Cancer 2010 Report, no conclusions can be reached for the evidence of fruits and vegetables consumption and breast cancer incidence^4^.

With a growing number of breast cancer survivors, some studies have investigated the association between consumption of VF and breast cancer survival^5–14^. The majority of these studies^6,7,9,11,12^ have reported a non-significant inverse association between VF intake and overall (OS) or breast cancer-specific survival (BCSS). However, two studies^8,10^ reported inverse associations of consumption of vegetables and fruits with all-cause mortality, and a trend for this relationship was observed in another study^5^. One study^10^ also found the inverse association with breast cancer-specific mortality. The 2014 report of the World Cancer Research Fund/American Institute for Cancer Research (WCRF/AICR) systematic analysis on surviving breast cancer^15^ showed that no significant associations were found between intake of VFs and breast cancer prognosis. The 2014 report presented results of studies published until 30^th^ June, 2012. The purpose of this study was to summarize results on the association between consumption of VFs and breast cancer survival. In this report, we updated the literatures search up to September 30, 2016 and performed quality assessment in detail. We conducted several subgroup analyses according to menopausal state, intake dose and adjustments; and specifically, we conducted analysis for intake of cruciferous vegetables (CVs).

## Results

### Study selection process

**Figure 1:**
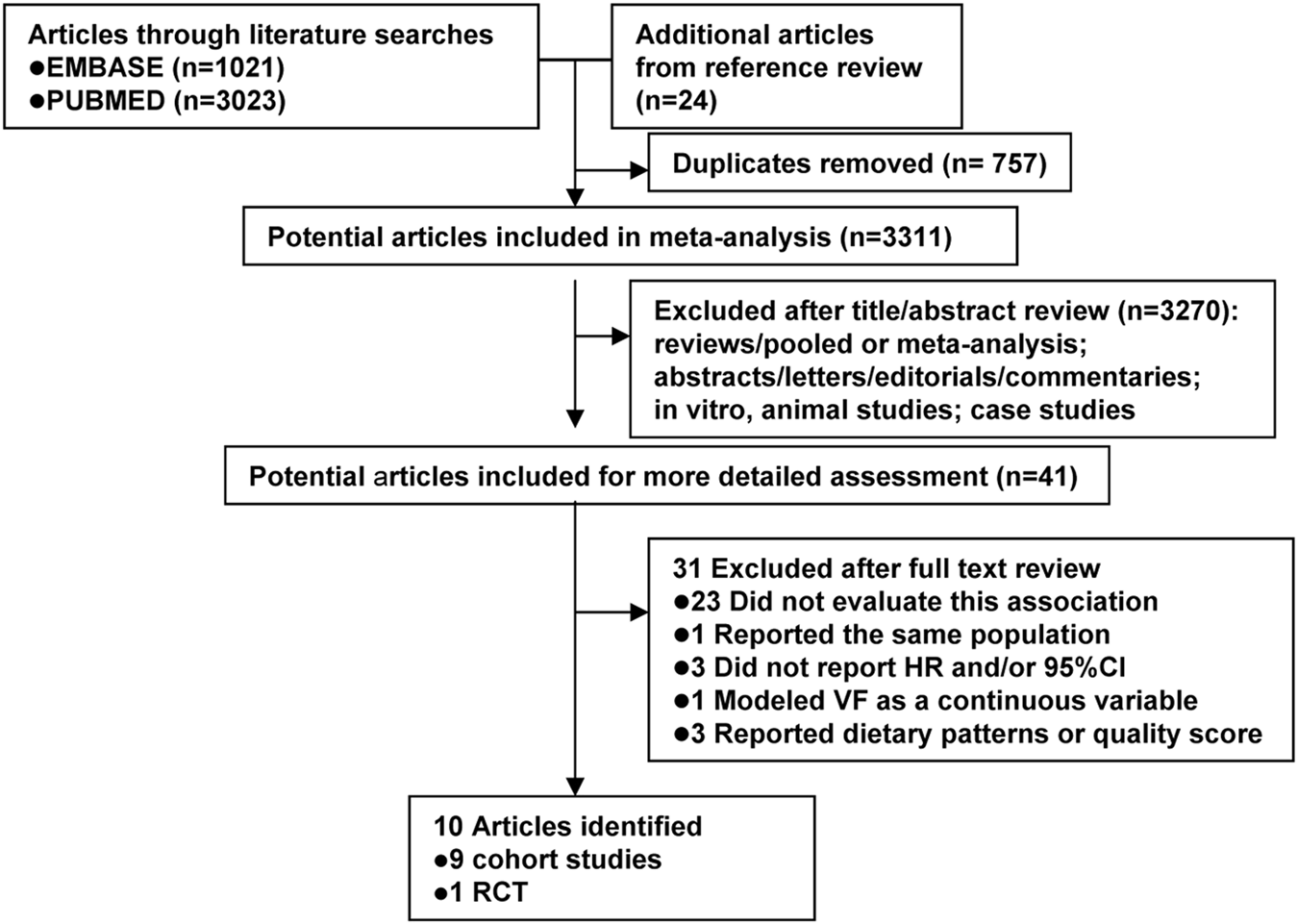
Flow diagram representing systematic literature search on vegetables and fruits intake and breast cancer survival.

The flow chart summarizing the process of study selection is shown in Fig. 1. According to the search criteria, we identified a total of 4,068 articles (3,023 from PubMed, 1,021 from EMBASE, and 24 from reference lists). Among these 4068 articles, 757 duplicates were identified and removed. After reviewing the title and abstract of the remaining 3,311 articles, 3,270 were excluded from the meta-analysis. Forty-one studies were evaluated in detail. After excluding studies that did not meet the inclusion criteria (see the methods section), a total of 10 articles were included in the meta-analysis (Table 1**)**. The most common reasons for exclusion were a lack of data on intake of VF or breast cancer prognosis (n = 23 studies). One study^16^ was excluded because its data duplicated in other articles. Another study^17^ modeled VF intake as a continuous variable. Three studies were excluded because no hazard ratios (HRs) and corresponding 95% confidence intervals (CIs) were reported or no information was used to calculate these estimates. Three studies^18–20^ were excluded because they reported dietary patterns or score.

**Table 1:** Characteristics of the included studies.

Author/year/Country	Cohort size Age, Follow-up	Diet assessment	Exposure (highest vs. lowest)	Primary event (***n***)	Adjustments
Holmes/1999/USA^5^	NHS	FFQ,	V: 4.2 vs. ≤2.12 servings/d	OS, n = 378	Age, diet interval, year of diagnosis, BMI, oral contraceptive use, menopausal status, postmenopausal hormone use, smoking, age at first birth and parity, number of metastatic lymph nodes, tumor size, caloric intake.
	N = 1982	After diagnosis	F: Q4 vs. Q1		
	Mean age, 54 years				
	Follow-up: 12.1 years				
Sauvaget/2003/UK^6^	LSS	Mailed	V: daily vs. 0–1/week	OS, n = 76	Age, radiation dose, city, BMI, smoking status, alcohol habits, education level, age at menarche, age at first birth, parity, breast-feeding, menopausal status, history of breast adenoma, family history
	Mean age, 56 years (range 34–103 years)	FFQ	F: daily vs. 0–1/week		
	Follow-up: 16 years	Before diagnosis			
Fink/2006/USA^7^	LIBCSP	Interview	VF: 46 + vs. 0–18 servings/wk	OS n = 186	Age, energy intake
	N = 1235	FFQ	V:24 + vs. 0–8 servings/wk	BCSS n = 125	
	invasive breast cancer	Before diagnosis	F: 24 + vs. 0–6 servings/wk		
	Age, 25–98 years				
	Follow-up: 1996–2004 (range)				
McEligot/2006/USA^8^	N = 516	Self-administered FFQ-100, Before diagnosis	V:3.1 vs. 0 servings/d	OS, n = 96	Age, stage of disease, BMI, parity, HRT, alcohol use, multivitamin use, energy intake
	Mean age, 64.8 years (9.3 years)		F: 2 vs. 0 servings/d		
	Follow-up: 6.6 years				
Pierce/2007/USA^9^	WHEL	dietary recalls telephone	VF: >8.92 vs. ≤ 4.94 servings/d	OS, n = 315	Age, tumor stage, clinic site, antiestrogen use, oophorectomy status
	RCT: N = 3088	After diagnosis	V: >4.8 vs. ≤2.55 servings/d		
	Mean age, 53.3 (8.9) years		F: >4.38 vs. ≤1.76 servings/d		
	early stage (I-IIIA)				
	Follow-up:7.3 years				
Dal Maso/2008/Italy^10^	N = 1453	in-person interviews	VF: <4 vs. >6 servings/d	OS, n = 503	Age, region of residence, year of diagnosis, TNM stage and ER/PR status
	62.7% TNM stage III-IV	FFQ-78		BCSS, n = 398	
	Median age, 55 years (range 23–74 years) Follow-up:12.6 years	Before diagnosis			
Buck/2011/German^12^	N = 2653 >80% non-metastasis	in-person interviews	V:183 vs 79 g/d	OS, n = 321	Age, tumor size, nodal status, metastasis, grade, ER/PR status, breast cancer detection type, diabetes, HRT, study centre, energy intake
	Median age, 63 years (range 50–74 years); Follow-up: 6.4 years	FFQ-176	F: 259 vs. 79 g/d	BCSS, n = 235	
		Before diagnosis			
Beasley/2011/USA^11^	CWLS, N = 4441	Mailed	V: 2.5 vs. 0.4 servings/d	OS, n = 525	Age, state of residence, menopausal status, smoking, stage, alcohol, HRT, interval between diagnosis and diet assessment, energy intake, breast cancer treatment, BMI, physical activity
	Mean age, 53.6 (range 20–79 years)	FFQ-126, After diagnosis	F: 2.5 vs. 0.1 servings/d	BCSS, n = 137	
	Follow-up: 5.5 years				
Nechuta/2013/USA^13^	ABCPP, N = 11390; Mean age, 51.1–64.5 years	FFQ, After diagnosis	CV: >78 vs. <39 g/d	OS, n = 1725	Age at diagnosis, ER/PR status, TNM stage, chemotherapy, surgery, radiotherapy, hormonal therapy, smoking, BMI, exercise, menopausal status, race/ethnicity, and education.
	Follow-up: 9.0 years				
McCullough/2016/USA^14^	CPS-II, N = 4452	Mailed	VF: T3 vs. T1	OS n = 1204	Age at diagnosis, diagnosis year, tumor stage, tumor grade, ER/PR, initial treatment, BMI, smoking status, physical activity, energy intake, dietary factors
	Mean age, 70.7 (7.2 years)	FFQ-68		BCSS n = 398	
	Follow-up: 9.8 years	Before and after diagnosis			

Abbreviations: V, vegetable; F, fruit; VF, vegetable and fruit; BMI, body mass index; LSS, Life Span Study; LIBCSP, Long Island Breast Cancer Study Project; WHEL, The Women’s Healthy Eating and Living Randomized Trial; CWLS, Collaborative Women’s Longevity Study; CPS-II, Cancer Prevention Study-II Nutrition Cohort; ABCPP, the After Breast Cancer Pooling Project.

### Characteristics of studies

Table 1 summarizes the characteristics of these 10 articles (9 cohort studies^5–8,10–14^ and 1 randomized control trial [RCT]^9^). Overall, 10 articles reported on OS^5–14^ and 5 on BCSS^7,10–12,14^. During a median follow-up duration of 6.6 years, there were a total of 5,329 total deaths (ranged from 76 to 1,725) and 1,293 (ranged from 125 to 398) breast cancer specific mortality cases among 31,210 women with breast cancer. Articles were published between 1999 and 2016. Studies were conducted in the following geographic regions: 7 from the United States, 3 from Europe (UK, Italy and Germany). Five studies^6–8,10,12^ collected information on dietary intake before diagnosis. In one study^7^, diet was assessed on average 3 months after diagnosis when 2/3 of the patients had not started chemotherapy treatment. One study^6^ assessed the average frequency of intake over the previous year, and two studies^8,10^ assessed dietary intake within 1 year of diagnosis. Four studies^5,9,11,13^ collected information on dietary intake after diagnosis, and one study^14^ before and after diagnosis. The quality scores of each study were summarized in Supplementary Table 1. The quality scores ranged from 7 to 9 for cohort studies and were 3 for one RCT study. All of included studies were of high quality (NOS score ≥7 and Jadad score ≥3).

### Pre-diagnostic dietary consumption and breast cancer OS

#### High vs. low analyses

**Figure 2:**
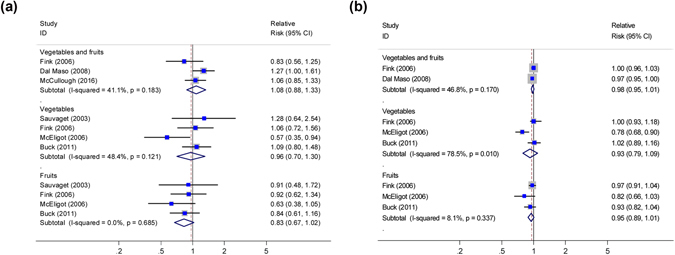
Relative risks for the association between pre-diagnostic intake of vegetables and fruits and all-cause mortality in breast cancer patients. (**a**) high *vs*. low analysis; (**b**) dose-response analysis for intake in increment of 1 serving/day. Squares indicated study-specific risk estimates (size of square reflects the study-statistical weight, i.e. inverse of variance); horizontal lines indicate 95% confidence intervals; diamond indicates summary relative risk estimate with its corresponding 95% confidence interval.

As shown in Fig. 2a, three studies^7,8,10^ reported an association between pre-diagnostic intake of vegetables and fruits combined and OS among breast cancer patients, and a non-significant association was found (summary HR = 1.08, 95% CI, 0.88–1.33; P_heterogeneity_ = 0.183, I^2^ = 41.1%). Four studies^6–8,12^ reported associations between pre-diagnostic intake of vegetables and fruits and OS, respectively, with a combined HR of 0.96 (95% CI, 0.71–1.30; P_heterogeneity_ = 0.121, I^2^ = 48.4%) for vegetables alone, and 0.83 (95% CI, 0.67–1.02; P_heterogeneity_ = 0.685, I^2^ = 0) for fruits alone.

#### Dose–response analyses

As shown in Fig. 2b, two studies^7,10^ were part of this dose-response analysis for the association between pre-diagnostic intake of vegetables and fruits and OS. The summary HR per 1 serving/day increment was 0.98 (95% CI: 0.95–1.01), with evidence of moderate heterogeneity (I^2^ = 46.8%, P_heterogeneity_ = 0.170). Three studies^7,8,12^ were included in the dose–response analysis for pre-diagnostic intake of vegetables and fruits and OS, respectively, with a summary HR of 0.93 (95% CI: 0.79–1.10; P_heterogeneity_ = 0.010, I^2^ = 78.5%) intake of vegetables only and 0.95 (95% CI: 0.89–1.01; P_heterogeneity_ = 0.337, I^2^ = 8.1%;) intake of fruits only.

### Post-diagnostic dietary consumption and breast cancer OS

#### High vs. low analyses

**Figure 3:**
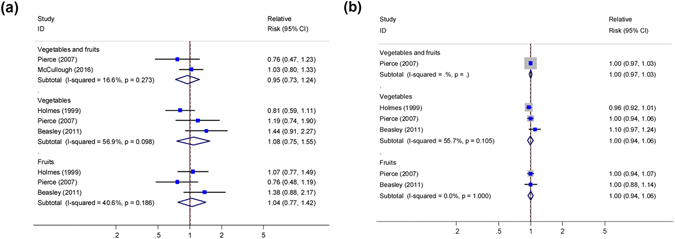
Relative risks for the association between post-diagnostic intake of vegetables and fruits and all-cause mortality in breast cancer patients. (**a**) high *vs*. low analysis; (**b**) dose-response analysis for intake in increment of 1 serving/day. Squares indicated study-specific risk estimates (size of square reflects the study-statistical weight, i.e. inverse of variance); horizontal lines indicate 95% confidence intervals; diamond indicates summary relative risk estimate with its corresponding 95% confidence interval.

As shown in Fig. 3a, two studies^9,14^ reported an association between post-diagnostic intake of vegetables and fruits combined and OS among breast cancer patients, and a non-significant association was found (summary HR = 0.95, 95% CI, 0.73–1.24; P_heterogeneity_ = 0.273, I^2^ = 16.6%). Three studies^5,9,11^ reported associations between post-diagnostic intake of vegetables and fruits and OS, respectively, with a combined HR of 1.08 (95% CI, 0.75–1.55; P_heterogeneity_ = 0.098, I^2^ = 56.9%) for vegetables alone, and 1.04 (95% CI, 0.77–1.42; P_heterogeneity_ = 0.186, I^2^ = 40.6%) for fruits alone.

### Dose–response analyses

As shown in Fig. 3b, one study^9^ was part of this dose-response analysis for the association between intake of vegetables and fruits and OS, with a HR per 1 serving/day increment of 1.00 (95% CI: 0.97–1.03). Three studies^5,9,11^ were included in the dose–response analysis for intake of vegetables only, with the summary HR of 1.00 (95% CI: 0.94–1.06; I^2^ = 55.7%, P_heterogeneity_ = 0.105). Two studies^9,11^ were included in the dose–response analysis for intake of fruits only, with the summary HR of 0.99 (95% CI: 0.97–1.02; I^2^ = 0, P_heterogeneity_ = 1.00; Fig. 3b).

#### Subgroup, meta-regression and sensitivity analyses

Subgroup and meta-regression analyses are presented in Table 2. Overall, no significant associations were observed between pre-diagnostic intake of vegetables and fruits and OS in all of the strata. For pre-diagnostic intake of vegetables alone, a stratified analysis by intake dose produced a non-significant summary HR for studies using low dose (summary HR = 1.12; 95% CI, 0.85–1.48) and high dose (summary HR = 0.79; 95% CI, 0.43–1.45). Combining studies with adjustments for tumor stages, treatment, BMI, smoking led to a non-significant association (for example: adjustments for tumor stages: summary HR = 0.81; 95% CI, 0.41–1.53; adjustments for treatment: summary HR = 1.28; 95% CI, 0.64–2.55). For pre-diagnostic intake of fruits alone, combining studies with adjustments for clinical tumor stage led to a borderline significant association (HR = 0.77, 95% CI: 0.59–1.01). Non- significant associations were observed for studies using low dose (HR = 0.92, 95% CI: 0.72–1.16) and high dose (HR = 0.79, 95% CI: 0.55–1.05).

**Table 2:** Stratified meta-analyses of vegetables and fruits intake and all cause mortality in breast cancer cases.

	Pre-diagnosis				Post-diagnosis			
	n	HR (95% CI)	P_h,_ I^2^ (%)	P_d_	n	HR (95% CI)	P_h,_ I^2^ (%)	P_d_
**Vegetables intake**								
All	4	0.96 (0.71–1.30)	0.121, 48.6		3	1.08 (0.75–1.55)	0.098, 56.9	
***Menopausal status***								
Pre-menopausal	1	1.40 (0.71–2.76)	—	0.408	0			
Postmenopausal	3	0.86 (0.59–1.25)	0.092, 58.1		0			
Intake dose*								
Low	2	1.12 (0.85–1.48)	0.676; 0	0.401	1	1.44 (0.91–2.27)	—	0.438
High	2	0.79 (0.43–1.45)	0.053; 73.4		2	0.94 (0.65–1.36)	0.184, 43.3	
***Adjustments***								
Clinical characteristics	2	0.81 (0.43–1.53)	0.029, 79.0	0.483	3	1.08 (0.75–1.55)	0.098, 56.9	—
Treatment	1	1.28 (0.64–2.55)	—	0.560	2	1.31 (0.95–1.82)	0.569, 0	0.286
BMI	1	1.28 (0.64–2.55)	—	0.560	2	1.05 (0.60–1.85)	0.042, 75.7	0.852
Smoking	1	1.28 (0.64–2.55)	—	0.560	2	1.05 (0.60–1.85)	0.042, 75.7	0.852
Physical activity	0				1	1.44 (0.91–2.27)	—	0.438
**Fruits intake**								
All	4	0.83 (0.67–1.02)	0.685, 0		3	1.04 (0.77–1.42)	0.186, 40.6	
***Menopausal status***								
Premenopausal	1	1.10 (0.48–2.52)	—	0.543	0			
Postmenopausal	3	0.80 (0.64–1.01)	0.582, 0		0			
Intake dose*								0.432
Low	2	0.85 (0.64–1.14)	0.826, 0	0.795	1	1.38 (0.88–2.17)	—	
High	2	0.79 (0.55–1.05)	0.245, 26.2		2	0.94 (0.68–1.30)	0.232, 29.9	
**Adjustments**								
Clinical characteristics	2	0.77 (0.59–1.02)	0.348, 0	0.517	3	1.04 (0.77–1.42)	0.186, 40.6	—
Treatment	1	0.91 (0.48–1.72)	—	0.790	2	1.03 (0.57–1.84)	0.068, 70.0	0.487
BMI	1	0.91 (0.48–1.72)	—	0.790	2	1.17 (0.90–1.53)	0.372, 0	0.355
Smoking	1	0.91 (0.48–1.72)	—	0.790	2	1.17 (0.90–1.53)	0.372, 0	0.355
Physical activity	0				1	1.38 (0.88–2.17)	—	0.432

^*^High, the highest level of vegetable/fruit intake: ≥3.1 servings/d; Low, the highest level of vegetable/fruit intake >1.8 servings/d.

**P**_**h**,_ P values for between-study heterogeneity; **P**_**d**,_ P values for the differences between subgroups.

Likely, no significant associations were observed between post-diagnostic intake of vegetables and fruits and OS in all of the strata. For post-diagnostic intake of vegetables alone, non-significant associations were observed for studies using low dose (HR = 1.44, 95% CI: 0.91–2.27) and high dose (HR = 0.94, 95% CI: 0.65–1.36). Combining studies with adjustments for tumor stages, treatment, BMI, smoking led to non-significant associations (For example: adjustments for treatment: summary HR = 1.31; 95% CI, 0.95–1.82; adjustments for BMI: summary HR = 1.05; 95% CI, 0.60–1.85).

Meta-regression analysis showed that no variables were significant factors for the association between pre- and post-diagnostic intake of vegetable and fruits and OS in women with breast cancer. In the leave-one-out sensitivity analysis, the pooled HR and 95% CI were not significantly altered (Supplementary Fig. 1A–D), which confirmed the robustness of the findings.

### Breast cancer-specific survival

**Figure 4:**
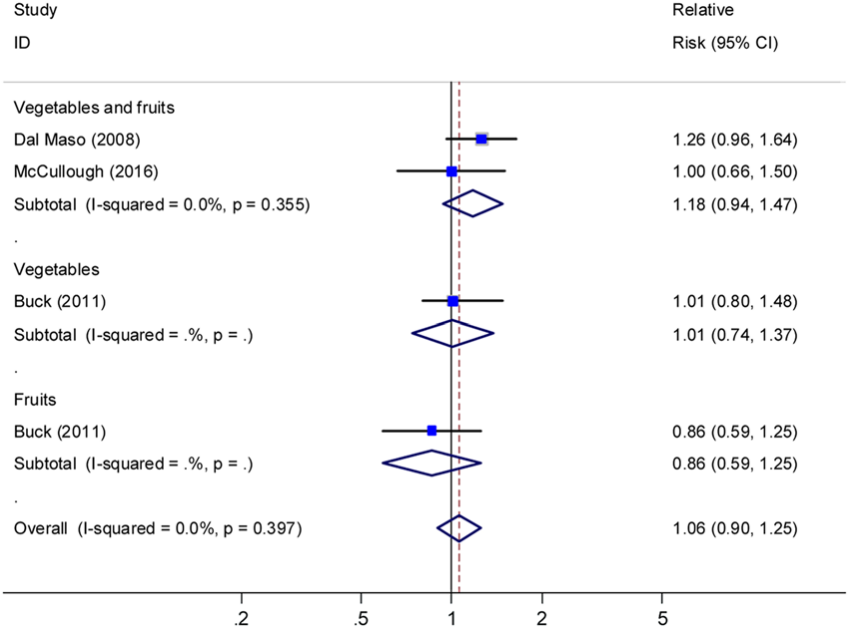
Relative risks for the association between pre-diagnostic intake of vegetables and fruits and breast cancer specific survival (high *vs*. low). Squares indicated study-specific risk estimates (size of square reflects the study-statistical weight, i.e. inverse of variance); horizontal lines indicate 95% confidence intervals; diamond indicates summary relative risk estimate with its corresponding 95% confidence interval.

Pooling two studies^10,14^ led to a null association between pre- diagnostic intake of vegetables and fruits combined and BCSS, with the summary HR of 1.18 (95% CI: 0.94–1.17; I^2^ = 0, Fig. 4). One studies^12^ presented null risk associations of BCSS for pre-diagnosis intake of vegetables and fruits, and another study^11^ for post-diagnosis intake of vegetables and fruits.

### CVs and breast cancer survival

**Figure 5:**
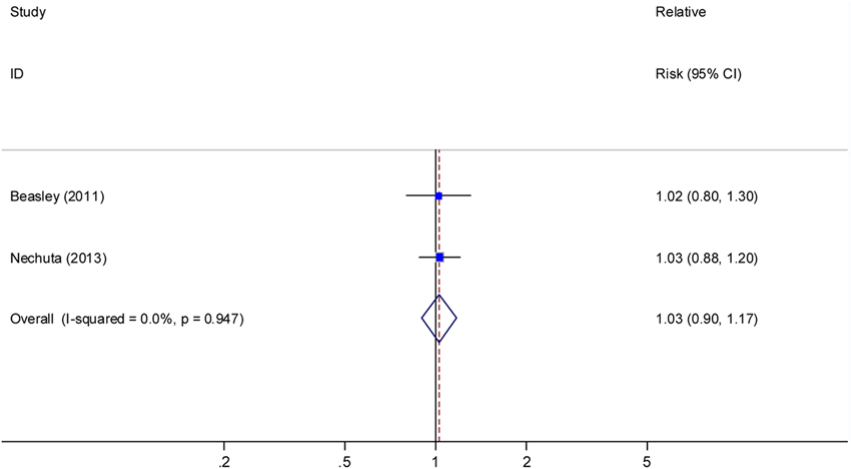
Relative risks for the association between post-diagnostic intake of cruciferous vegetables and breast cancer overall survival (high *vs*. low). Squares indicated study-specific risk estimates (size of square reflects the study-statistical weight, i.e. inverse of variance); horizontal lines indicate 95% confidence intervals; diamond indicates summary relative risk estimate with its corresponding 95% confidence interval.

Two studies^11,13^ presented results for OS with post-diagnosis intake of CVs, with the summary HR (high vs. low) of 1.03 (95% CI, 0.90–1.00; I^2^ = 0, Fig. 5). One study^11^ presented no significant association between post-diagnosis intake of CVs and BCSS (HR = 0.95, 95% CI: 0.59–1.54; P for trend = 0.86). Another study^7^ showed a null association between pre-diagnosis intake of CVs and OS for both pre- and post-menopausal women with breast cancer.

## Discussion

We have evaluated the association between intake of vegetables and fruits and breast cancer outcomes using a meta-analysis of previously published studies. Overall, results of this meta-analysis suggest that intake of vegetables and fruit is not significantly associated with total and specific death from breast cancer. In subgroup meta-analysis, a borderline reduced risk of total death was observed for pre-diagnosis intake of fruits alone among women with breast cancer. In addition, intake of cruciferous vegetables was not associated with death from breast cancer.

As early diagnosis and treatments for breast cancer improve, breast cancer prognosis becomes good in most of the developed countries, with a five-year survival rate of more than 85% in women with localized and regional disease^21^. Lifestyle factors become increasingly important, which may help to lower the risk of dying from the disease. However, researches exploring the science behind surviving breast cancer are relatively scarce. The evidence from well-conducted RCTs may be stronger than that from cohort studies. The Women’s Healthy Eating and Living (WHEL) RCT^9^ observed that women in the intervention arm who consumed 5 vegetable servings, 16 oz vegetable juice, 3 fruit servings, 15–20% of energy, and 30 g fiber per day did not lower risk of recurrence and death from breast cancer. A few cohort studies^5–8,10–14^ have examined the effects of dietary factors on mortality outcomes in breast cancer survivors with inconsistent results. The 2014 breast cancer survivors report from WCRF/AICR^15^ suggested that a higher consumption of VF may not be related to surviving breast cancer.

In the current study, we found pre-diagnosis intake of fruits alone was associated with borderline decrease in risk of total death among women with breast cancer, which also were confirmed according to dose-response analysis. However, only four studies were included for pre-diagnosis intake of fruits alone among women with breast cancer, we should be caution that this relationship was only due to chance, because we could not exclude a type **Ι** error.

Several potential mechanisms might explain a protective effect of vegetables and fruits on breast cancer prognosis, which may be through high intakes of fiber, micronutrients, including vitamins C, E, folate and carotenoids^22,23^. In cell culture and animal studies, carotenoids have retinoid-like effects on cellular differentiation and apoptosis and also exhibit inhibitory effects on mammary cell growth^24,25^. Vitamin C exhibits antioxidant actions including the neutralisation of free radicals, which may impact cancer progression^26,27^. Furthermore, dietary intake of folate was associated with a reduced death by inducing incorporation of uracil into DNA and inducing altered gene expression of critical tumor suppressor and proto-oncogenes *via* its role in DNA methylation^28–30^. Epidemiological studies^31^ and clinical trial^32^ have suggested that a high fiber intake was related to reductions in blood levels of estrogen. Recent study has indicated that higher preoperative serum estrogen level had a negative prognostic effect in postmenopausal women with breast cancer, especially in the ER-negative subgroup^33^.

Strengths of this analysis include independent search and data abstraction by two researchers experienced in conducting systematic reviews; performing quality assessment in detail; exploring the aforementioned associations according to pre- and post-diagnosis, respectively; examining both high vs. low and dose–response relationships; conducting several subgroup and meta-regression analyses.

This meta-analysis has limitations. First, some misclassification of vegetables and fruits intake is likely. To reduce the errors in assessment of dietary intake, we included studies evaluating all fruits or vegetables. However, populations from different regions, ethnicities and time periods may have differences in their classifications and types consumed. Dietary intake was assessed with a food frequency questionnaire (FFQ) in all but one study which could have introduced measurement errors due to poor recall. One study^9^ included in our meta-analysis used 24 h-diet recalls, which are often considered more robust dietary assessment methods. Previous studies^34,35^ have suggested the relatively low correlation in report their intake of vegetables or fruit (Spearman’s correlation coefficients of 0.6 for fruit consumption and 0.4 for vegetable consumption). However, it was assessed before the outcome of interest (mortality) and thus is most likely non-differential. In addition, analyses of the highest vs. the lowest intake are limited because they do not account for true differences among studies. For example, the definition of the lowest exposure ranged from 0^8^ to <2.55^9^ servings/d of vegetables intake, and the highest intake levels of vegetables ranged from 183 g/d (about 1.8 servings/d)^12^ to >4.8^9^ servings/d. Although statistically not significant, subgroup analyses demonstrated that OS was improved when the meta-analysis was restricted to high dose intakes of vegetables/fruits subgroup. The non-significant results might be due to low statistical power since only two studies were included in the high dose categories. More studies with larger sample size and long duration of follow-up are warranted.

Second, since not all potential confounders were adjusted for in the individual studies, there remains the possibility of residual or unmeasured confounding. For example, not all studies were adjusted for clinical characteristics and cancer treatments, both of which are the most important prognostic factors. Our meta-analysis found no associations between intake of vegetable and fruit and total mortality among breast cancer across studies whether or not with adjustments for clinical characteristics. In one of the included studies^5^, the authors found there was significant association with intakes of vegetables and reduced mortality among women without metastatic lymph nodes (HR = 0.62, 95% CI: 0.36–1.07, P for trend = 0.02), but no significant association among women with metastatic lymph nodes (HR = 0.90, 95% CI: 0.60–1.33, p for trend = 0.53). Therefore, further investigations on vegetables and fruits intake and breast cancer prognosis by clinical tumor characteristics are needed. On the other hand, disease detection (clinical or screening) is another potential confounder because screen-detected cases show lower stage and longer survival than cancer found incidentally^36,37^. However, data on mode of detection were not available in the study.

Higher consumption of fruit and vegetables may be a marker for generally “healthier” dietary and lifestyle patterns, such as drinking less alcohol, a lower prevalence of overweight/obesity and cigarette smoking, and being physically active^38,39^. A healthy lifestyle may lower the occurrence of breast cancer. Although the evidence available is limited^15,40–42^, cigarette smoking, overweight/obesity and being physical inactivity (before and after a diagnosis) are suggested to be linked to increased total and specific cause of mortality in women with breast cancer. Stratified and meta-regression analyses indicated that risk of death for consumption of fruits and vegetables were not significantly changed by adjustment for BMI, smoking, and physical activity. However, only a few studies employed adjustments for these factors. Hence, we cannot fully exclude the possibility of residual confounding.

Third, studies included in this meta-analysis were major conducted in Western countries (7 from the United States, 3 from Europe) and in middle-aged and older person; so, the results should be extrapolated to other populations with caution. In addition, the number of studies included in the meta-analysis was small, reducing the power of the meta-analysis and thereby reduced the generalizability of our findings. The clinical behavior of ER-positive and ER-negative breast cancers differ significantly. We did not carry out stratified analysis by ER/progesterone receptor (PR) status because few studies provided the necessary data. One study^10^ reported that high vegetables and fruits intake significantly improved disease prognosis among ER/PR-negative breast cancer patients. Therefore, further investigations on vegetables and fruits intake and breast cancer prognosis by ER/PR status are needed.

Finally, publication bias should always be considered in a meta-analysis of published data, because the results of large cohort studies (even null results) are more likely to be published. However, we could not perform such an analysis due to the small studies included.

In conclusion, intake of vegetables alone was not significantly associated with overall survival of breast cancer, however, intake of fruit alone might has, if any, a small effect on the overall survival among women with breast cancer. Considering the limited number of included studies, further studies with larger sample size, well-controlled confounding factors, long enough follow-up time, and more accurate assessment of dietary intake are warranted.

## Methods

### Search strategy and study selection

We (HJJ and GYT) conducted a literature search using the MEDLINE (http://www.ncbi.nlm.nih.gov/pubmed/, from January 1, 1966) and EMBASE (http://www.embase.com/, from January 1, 1974) databases. The following search terms were used: 1) breast cancer OR breast neoplasm OR breast carcinoma; 2) mortality OR survival OR prognosis OR disease progression OR outcome OR death; and 3) nutrition OR diet OR lifestyle OR fruit OR vegetable. All results were updated on September 30, 2016. In addition, we carried out manual searches of the reference lists from retrieved articles to identify additional studies. Only full-length original articles published in English were considered, and no attempt was made to include abstracts or unpublished data. This meta-analysis was planned, conducted, and reported according to the preferred reporting items for systematic reviews and meta-analyses (PRISMA) statement^28^.

In the present meta-analysis, we included the studies evaluating fruit and/or vegetable groups classified as “all” or “total”. We did not include exposures presented as raw vegetables, green-yellow vegetables, cooked vegetables, green leaf vegetables, other vegetables, citrus fruit, or other specific types of fruits. But we included studies which reported “fresh vegetables” or “fresh fruit”, because fresh vegetables or fruit accounts for a very high proportion of the total consumption^43^.

To be included in the meta-analysis, the following criteria had to be met: (1) the exposure of interest was consumption of vegetable and/or fruits assessed before or after diagnosis (≥12 months); (2) the outcome of interest was BCSS or OS among women with breast cancer; and (3) reported HRs (95% CIs) or information to calculate these estimates (e.g., the number of cases and person-years of follow-up or number of noncases). Two authors (HJJ and GYT) independently assessed titles and abstracts of the references identified by the search strategy according to the selection criteria. Any differences in opinion with regard to eligibility were resolved by a third investigator (ZSJ). When unclear from the title or abstract whether the paper fulfilled the criteria, or when there is disparity among authors, a full text copy were obtained. We did not assess breast cancer recurrence as endpoints of the meta-analysis, given that the definition used for recurrence varied across studies. We excluded studies assessing the association for nutrients provided by vegetables and fruit, such as fiber, carotenoids and vitamin C. Case reports, reviews, animal studies, and *in-vitro* studies were also excluded. When two or more studies presented possible overlap, the one of better quality or with more detailed data were included.

### Data extraction

The following data were extracted using a standardized data collection form: author’s name, publication year, country or region, name of study, number of participants, age, number of breast cancer cases and number of deaths (OS and/or BCSS), timing of data collection (before or after diagnosis) and type of diet assessment, range of exposure and adjustments. We used the maximally adjusted results when several risk estimates with various adjustments were reported. Data were extracted independently by two investigators (HJJ and GYT). Discrepancies were resolved by discussion and repeated examination of the studies to reach a consensus.

### Quality assessment of individual studies

For the assessment of the study quality of cohort studies, we used the Newcastle-Ottawa Scale (NOS) checklist^44^, which assesses the quality of case control and cohort studies against three parameter: selection (four items, with each being awarded one star), comparability (one item, which can be awarded up to two stars), and exposure/outcome (three items, with each being awarded one star). A score of ≥7 stars is indicative of a high quality study. For the assessment of the study quality of one RCT^9^, we used the five-point Jadad scale of randomisation (0 or 1), double-blinding (0, 1 or 2), treatment of withdrawals and dropouts (0 or 1), and allocation concealment (0 or 1)^45,46^. Studies were categorized as low quality (score ≤ 2) or high quality (score at least 3)^46,47^.

### Statistical analysis

All statistical analyses were performed using STATA, version 11.0 (STATA, College Station, TX, USA) statistical software. A two-tailed P value of <0.05 represents significance. The primary outcome analyzed was OS, and another point of interest was BCSS. Adjusted HR estimates were pooled for the highest vs. lowest level and linear dose-responses using random effects meta-analysis, which considers both within- and between-study variation and provides more conservative estimates than a fixed-effects model^48^. The study from Dal Maso *et al*.^10^ did not use the lowest category as a reference, we therefore recalculated the RR and its 95% CI. We also reported results for intake of CVs. When estimates were available specifically for both pre- and post-menopausal women with breast cancer^7^, they were combined according to a fixed effects model to obtain an overall estimate. The summary measures were presented as forest plots where the size of data markers (squares) corresponds to the inverse of the variance of the natural logarithm of RR from each study, and the diamond indicates pooled RR.

Heterogeneity among articles was estimated using the *I*^2^-statistics and *p* values associated with Q statistics. P value of <0.10 represents statistically significant heterogeneity. *I*^2^ values explain the amount of total variation among studies that is due to heterogeneity. *I*^2^ value of >50% signify severe heterogeneity while a value of <25% represents no significant heterogeneity^49^. We explored heterogeneity between studies using three strategies. First, stratified analyses were carried out to estimate the effects of the following variables on outcomes: intake dose (the highest level of vegetable intake: ≥3.1 servings/d vs. >1.8 servings/d), menopausal status, confounding including clinical tumor stage, receiving treatment, BMI, physical activity and cigarette smoking. Because of the small number of studies reporting on BCSS, the stratified analyses were carried out only for OS. Second, we carried out stepwise meta-regression analyses. Third, we re-ran the meta-analysis removing studies once at a time to determine whether a particular study accounted for the heterogeneity.

The dose–response results in the forest plots are presented in one serving/day increment, which was according to the method of generalized least-squares trend estimation (GLST) developed by Greenland and Orsini^50,51^. Dose–response analysis is performed on the basis of the data for categories of intake levels on median dose, number of cases and participants and adjusted logarithm of the HR with its SE. Lack of this information led us to estimate the dose-response slopes using variance-weighted least squares (vwls) regression analysis^50,51^. We then combined the GLST-estimated study-specific slopes with studies that reported slope estimates to derive an overall average slope. This analysis was restricted to the studies reporting three or more exposure levels. In the absence of such median intake value, we used the midpoint of each category. For an open-ended upper category of intake, we assumed the size of the interval to be the same as the previous category. If the lowest category was open-ended, the lower boundary was assumed to be zero. When studies used different measurement units (e.g. grams per day), we standardized fruits and vegetables intake into servings per day using a standard portion size of 106 g^52^. Publication bias was not evaluated due to the small number of studies included.

## Electronic supplementary material

supple.
