# Author Correction: Consumption of vegetables and fruits and breast cancer survival: a systematic review and meta-analysis

**DOI:** 10.1038/s41598-018-26960-x

**Published:** 2018-06-01

**Authors:** Juanjuan He, Yuanting Gu, Shaojin Zhang

**Affiliations:** 1 Department of Breast Surgery, the First Affiliated Hospital, Zhengzhou University, Zhengzhou, Henan Province China; 2 Department of Urology Surgery, the First Affiliated Hospital, Zhengzhou University, Zhengzhou, Henan Province China

Correction to: *Scientific Reports* 10.1038/s41598-017-00635-5, published online 04 April 2017

The original version of this Article contained a typographical error in the spelling of the author Shaojin Zhang, which was incorrectly given as Shaojing Zhang. This has now been corrected in the PDF and HTML versions of the Article and in the accompanying Supplementary file.

