# Supplementary material for: Consumption of vegetables and fruits and breast cancer survival: a systematic review and meta-analysis
Source: Sci Rep. 2017 Apr 4;7:599. doi: 10.1038/s41598-017-00635-5 (PMC5428797; doi:10.1038/s41598-017-00635-5)
Supplement: Supplementary file 1 — supple. [file 41598_2017_635_MOESM1_ESM.doc]

**Consumption of vegetables and fruits and breast cancer survival: a meta-analysis**

Juanjuan He^1^, Yuanting Gu^1^, Shaojin Zhang^2^

A

**B**

**C**

**D**

**Supplementary Figure 1.** Influence of removing studies one by one on all-cause mortality in breast cancer. **(A)** pre-diagnostic intake of vegetables; (B) pre-diagnostic intake of fruits; **(C)** post-diagnostic intake of vegetables; (D) post-diagnostic intake of fruits... Circles are effect estimates and horizontal dotted lines were 95% confidence intervals for meta-analysis of the remained studies; the vertical line in the center is the pooled effect estimate for all studies.
